# E2F8 Induces Cell Proliferation and Invasion through the Epithelial–Mesenchymal Transition and Notch Signaling Pathways in Ovarian Cancer

**DOI:** 10.3390/ijms21165813

**Published:** 2020-08-13

**Authors:** Kyung Jin Eoh, Hee Jung Kim, Jong Woo Lee, Lee Kyung Kim, Sun-Ae Park, Hyun-Soo Kim, Young Tae Kim, Peter J. Koo

**Affiliations:** 1 Department of Obstetrics and Gynecology, Yonsei University College of Medicine, Yongin Severance Hospital, Yongin 16995, Korea; kjeoh2030@yuhs.ac; 2 Section of Medical Oncology, Department of Internal Medicine, Yale Comprehensive Cancer Center, Yale School of Medicine, Yale University, New Haven, CT 06520, USA; jongwoo.lee@yale.edu; 3 Laboratory of Pharmacoimmunology, Integrated Research Institute of Pharmaceutical Sciences, College of Pharmacy, The Catholic University of Korea, Bucheon 14662, Korea; hjk0114@hanmail.net (H.J.K.); 1202dlruddl@naver.com (L.K.K.); tjsdo37@yuhs.ac (S.-A.P.); 4 Division of Gynecologic Oncology, Department of Obstetrics and Gynecology, Institute of Women’s Life Medical Science, Severance Hospital, Yonsei University College of Medicine, Seoul 03722, Korea; 5 Department of Pathology and Translational Genomics, Samsung Medical Center, Sungkyunkwan University School of Medicine, Seoul 06351, Korea; hyunsookim@yuhs.ac

**Keywords:** E2F8, invasion, migration, ovarian cancer, epithelial–mesenchymal transition, Notch signaling

## Abstract

Background: Despite the recent research implicating E2F8 (E2F Transcription Factor 8) in cancer, the role of E2F8 in the progression of ovarian cancer has remained unclear. Hence, we explored the bio-functional effects of E2F8 knockdown on ovarian cancer cell lines in vitro and in vivo. Methods: The expression of E2F8 was compared between ovarian cancer and noncancer tissues, and its association with the progression-free survival of ovarian cancer patients was analyzed. To demonstrate the function of E2F8 in cell proliferation, migration, and invasion, we employed RNA interference to suppress E2F8 expression in ovarian cancer cell lines. Finally, the effect of E2F8 knockdown was investigated in a xenograft mouse model of ovarian cancer. Results: Ovarian cancer tissue exhibited significantly higher E2F8 expression compared to that of normal ovarian tissue. Clinical data showed that E2F8 was a significant predictor of progression-free survival. Moreover, the prognosis of the ovarian cancer patients with high E2F8 expression was poorer than that of the patients with low E2F8 expression. In vitro experiments using E2F8-knockdown ovarian cancer cell lines demonstrated that E2F8 knockdown inhibited cell proliferation, migration, and tumor invasion. Additionally, E2F8 was a potent inducer and modulator of the expression of epithelial–mesenchymal transition and Notch signaling pathway-related markers. We confirmed the function of E2F8 in vivo, signifying that E2F8 knockdown was significantly correlated with reduced tumor size and weight. Conclusions: Our findings indicate that E2F8 is highly correlated with ovarian cancer progression. Hence, E2F8 can be utilized as a prognostic marker and therapeutic target against ovarian malignancy.

## 1. Introduction

Ovarian cancer is the leading cause of gynecologic cancer-related mortality, owing to its aggressive metastasis, recurrence, and drug resistance. Although primary treatment of ovarian cancer is well-established with the combination of complete cytoreductive surgery and courses of platinum-based intravenous chemotherapy, the five-year survival rate is only about 30%, and therefore, an effective alternative strategy is needed to overcome tumor invasiveness and progression [1].

The E2F family of transcription factors is known to be involved in the regulation of the cell cycle as well as in cell proliferation, differentiation, DNA repair, and apoptosis [2,3,4]. Among the eight members of the E2F family, E2F1-3a is the transcriptional activator, whereas E2F3b-7 is the inhibitor of the transcription of downstream genes [5,6,7]. The functional role of E2F transcription factor 8 (E2F8), whose discovery as a member is relatively recent, remains uncertain.

Considering in vivo models, E2F8 is reportedly essential in embryonic development [8,9], angiogenesis [10], and lymphangiogenesis [11]. In particular, recent literature has shown that the expression of E2F8 is noticeably enhanced in multiple carcinomas, including lung cancer, breast cancer, and hepatocellular carcinoma [12,13,14], implying that E2F8 is involved in oncogenesis and cancer progression. However, the biological role of E2F8 in the progression, particularly of ovarian cancer and its clinical implications, remains to be elucidated [15,16]. Epithelial–mesenchymal transition (EMT) is an early event in the process of tumor invasion and metastasis [17]. Several studies have shown that it is widely recognized that the E2Fs signal EMT, invasion, and metastasis. For example, it has been shown that miR-30a-5p decreased EMT and metastasis in gallbladder carcinoma cells by targeting E2F7 [18]. E2F7 and E2F8 have also been shown to induce angiogenesis, which facilitates metastasis, by interacting with HIF1and regulating VEGFA [10]. Recent reports showed that E2F1 promotes EMT by regulating ZEB2 in small lung cancer [19]. Our previous research showed that E2F8 was also involved in invasion and metastasis by EMT in cervical cancer [20]. These results showed that E2F8 is closely associated with EMT, but the detailed mechanism of EMT regulation by E2F8 in ovarian cancer is unknown. Additionally, the Notch signal pathway has been found to be a key regulator in the induction of EMT [21,22,23]. Notch activation in endothelial cells results in morphological, phenotypic, and functional changes consistent with mesenchymal transformation. Therefore, it is believed that Notch-induced EMT is restricted to cells expressing activated Notch.

Fewer studies have been devoted to studying the role of E2F8 and the Notch signal pathway in EMT.

Hence, we aimed to investigate the involvement of E2F8 in the progression of ovarian cancer. Specifically, we sought to characterize the expression of E2F8 in ovarian cancer tissues and cell lines, to describe its relationship with the cancer prognosis, and to determine the effects of E2F8 knockdown on the proliferation, invasion, and migration of ovarian cancer cells as well as on tumor growth and on the EMT and Notch signaling pathways associated with the cancer.

## 2. Results

### 2.1. E2F8 Expression Elevated in Ovarian Cancer and Correlated with Poor Prognosis

**Figure 1:**
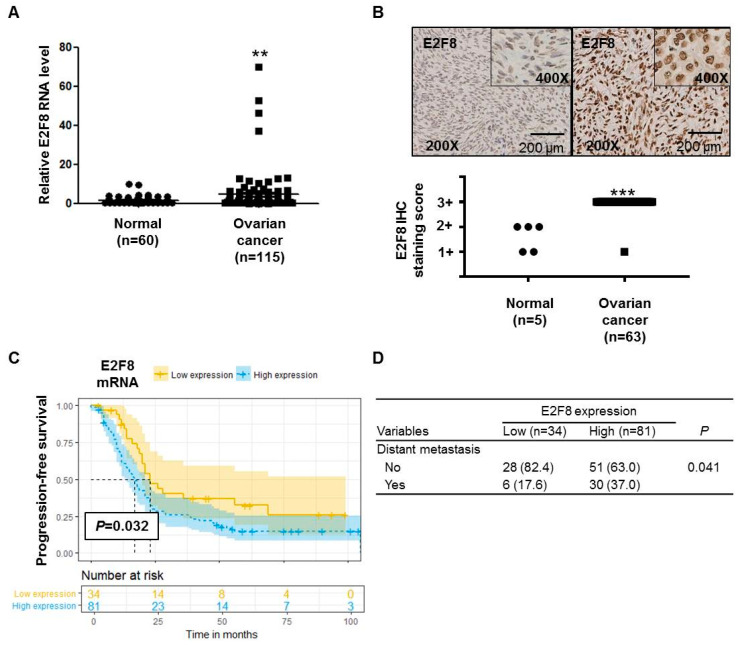
E2F8 expression in ovarian cancer and noncancer tissues and the correlation with prognosis. (**A**) E2F8 expression in ovarian cancer tissues (*n* = 115) and noncancerous tissues (*n* = 60). (**B**) Representative tissue microarray-based immunohistochemical analysis of E2F8 expression in normal and ovarian cancer tissues. Immunohistochemical analysis of E2F8 protein expression showing a significant difference in E2F8 protein expression between 63 ovarian cancer tissue samples and 5 normal ovarian tissue samples. (**C**) Progression-free survival in ovarian cancer patients with high and low E2F8 expression. (**D**) Comparison of the patient number shown to have distant metastasis according to E2F8 expression. ** *p* < 0.01, *** *p* < 0.001 vs. nontumor control.

The *E2F8* mRNA expression was significantly higher (*p* = 0.0078) in ovarian cancer tissues (*n* = 115) than in noncancerous tissues (*n* = 60) (Figure 1A). The IHC analysis determined that 63 ovarian cancer tissue samples showed more increased E2F8 expression than 5 normal ovarian tissue samples (*p* < 0.001) (Figure 1B). Moreover, the TMA slide-based IHC analysis showed stronger nuclear E2F8 immunoreactivities in the ovarian cancer tissues than in the normal ovarian tissues (Figure 1B).

We compared the characteristics of patients with high E2F8 expression (*n* = 81) with those with low E2F8 expression (*n* = 34) (Supplementary Table S1). There were no significant differences in several clinical variables, including age, the International Federation of Gynecology and Obstetrics (FIGO)-classified stage, tumor histology, grade, preoperational CA-125, and residual disease after cytoreductive surgery. As a result, there were no significant differences in the FIGO stage (Supplementary Table S1). However, the proportion of patients with distant metastasis was significantly higher among the patients with high E2F8 expression, compared to those with low E2F8 expression *(p =* 0.041) (Figure 1D).

The Kaplan–Meier survival analysis demonstrated that the ovarian cancer patients with high E2F8 levels had poorer progression-free survival compared to that of the patients with low E2F8 levels (*p* = 0.032) (Figure 1C). Moreover, according to the univariate and multivariate analyses using the Cox proportional hazards model, the E2F8 expression was a significant factor in predicting poor progression-free survival (univariate hazard ratio (HR): 1.887, 95% confidence interval (CI): 1.073–3.318, *p* = 0.028; multivariate HR: 1.590, 95% CI: 1.010–2.500, *p* = 0.15), along with well-known prognostic factors such as residual disease and the FIGO stage (Table 1).

### 2.2. E2F8 Expression Elevated in Ovarian Cancer Cell Lines and Correlated with Cell Proliferation

**Figure 2:**
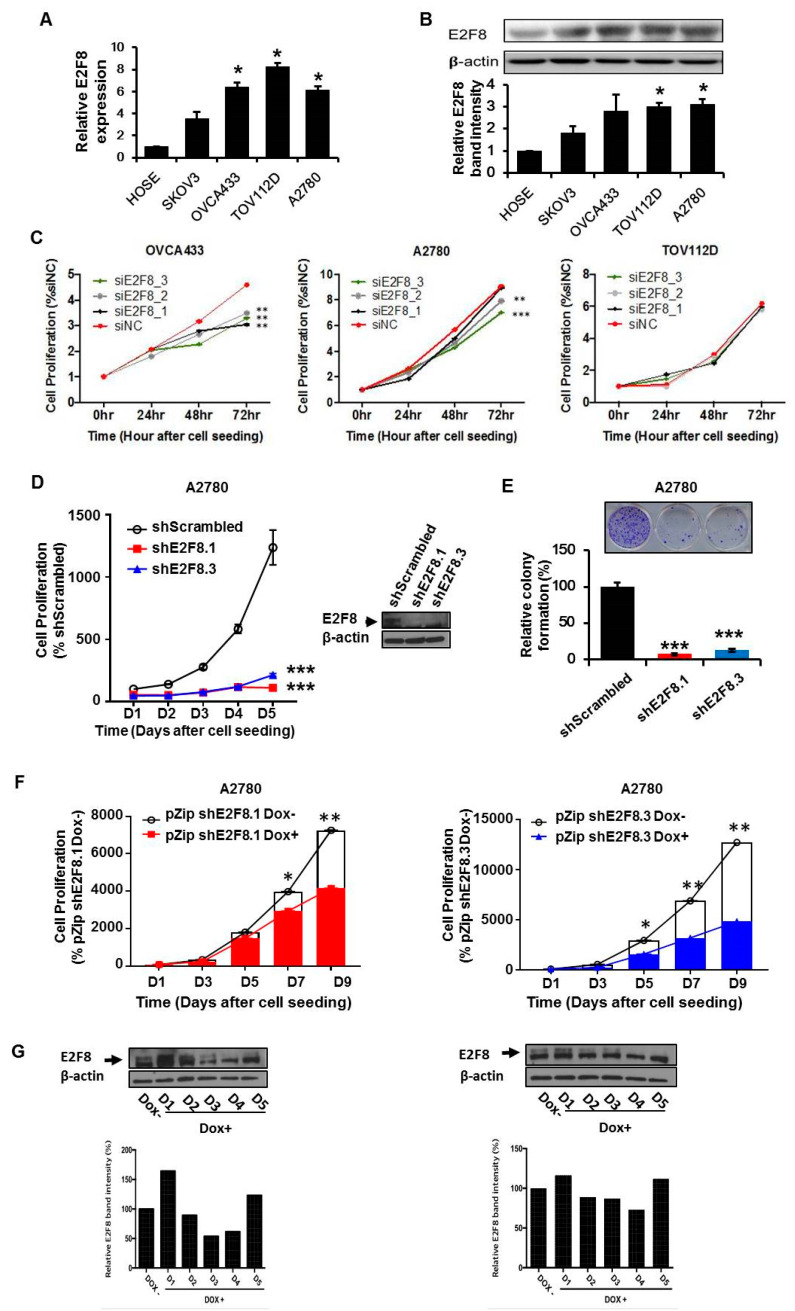
E2F8 expression in ovarian cancer cell lines and correlation with suppressed cell proliferation. (**A**,**B**) The knockdown validation data are shown in Supplement Figure 1. Expression levels of E2F8 mRNA and protein in some ovarian cancer cell lines (OVCA433, TOV112D, and A2780) and normal control cells. **(C**) The effect of E2F8-targeting siRNA (siE2F8)-transfection on cell proliferation in multiple cell lines. **(D**) Proliferation of shRNAs targeting E2F8 (shE2F8)-transfected A2780 cell line. (**E**) Colony formation in E2F8-knockdown A2780 cells. (**F**) Proliferation of doxycycline-inducible shE2F8.1- or shE2F8.3 -expressing stable A2780 cell lines in the presence or absence of doxycycline. (**G**) Doxycycline-inducible shE2F8.1- and shE2F8.3-transfected stable A2780 cell line was established. After treatment with doxycycline, protein lysates were obtained daily from day 1 to 5, and E2F8 protein expression was assessed * *p* < 0.05, ** *p* < 0.01, *** *p* < 0.001.

We determined that the expression of E2F8 at the mRNA and protein levels was significantly higher in the ovarian cancer cell lines OVCA433, TOV112D, and A2780 than in the normal control cells (Figure 2A,B). Then, we examined the effects of E2F8 on the ovarian cancer cells. Using E2F8-targeting siRNA (siE2F8), E2F8 was knocked down in the OVCA433, TOV112D, and A2780 cell lines. The knockdown is validated by qRT-PCR in Supplementary Figure S1. Among the three cell lines, the siE2F8-transfected OVCA433 and A2780 cells showed significant suppression of cell proliferation (Figure 2C). Considering the cell proliferation results, the A2780 cell line was used in the subsequent experiments involving shRNAs targeting E2F8 (shE2F8) transfection and in vivo analysis. The A2780 cell line was transfected with two different shE2F8 molecules (shE2F8.1 and shE2F8.3), and the knockdown effects were validated at the protein level (Figure 2D). The shE2F8-transfected A2780 cells also exhibited downregulation of cell proliferation and colony formation (Figure 2D,E).

The doxycycline-inducible shE2F8.1- and shE2F8.3-transfected stable A2780 cell lines were established. After treatment with doxycycline, the protein lysates were obtained daily from day 1 to 5. By day 4, E2F8 protein expression had been abrogated (Figure 2G). The proliferation of the doxycycline-inducible shE2F8.1- or shE2F8.3-expressing stable A2780 cell lines was compared between the presence and absence of doxycycline. The inhibition of cell proliferation by E2F8 knockdown was statistically significant beginning day 7 and 5 in the A2780 cell lines transfected with doxycycline-inducible shE2F8.1 and shE2F8.3, respectively (Figure 2F).

### 2.3. E2F8 Knockdown Inhibits Invasion and Migration of Ovarian Cancer Cells

**Figure 3:**
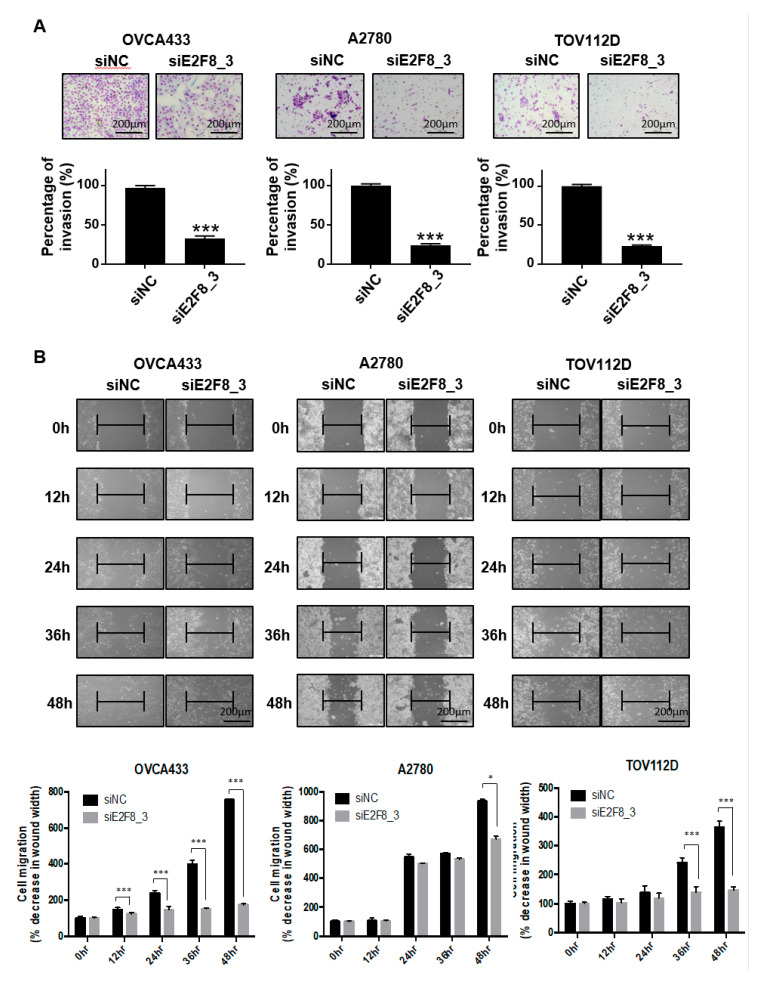
Knockdown of E2F8 inhibits the invasion and migration of OVCA433, A2780, and TOV112D cells. (**A**) Matrigel invasion assay was used to determine invasion after 48 h. (**B**) Wound-healing assay was used to determine migration in siE2F8-transfected OVCA433, A2780, and TOV112D cells (×200). This assay was performed in triplicate. Data are the mean ± SD. * *p* < 0.05, *** *p* < 0.001 vs. negative control siRNA [negative control siRNA (siNC)].

As shown in Figure 1D, the upregulation of E2F8 was significantly associated with distant metastasis in the ovarian cancer patients (*p* = 0.041). We therefore investigated whether E2F8 is related to the invasion and migration of ovarian cancer cells. A Matrigel invasion assay was utilized to assess the invasion after 48 h. The E2F8-knockdown ovarian cancer cell lines OVCA433, A2780, and TOV112D showed significant decreases in cell invasion, in comparison with the control cells (Figure 3A). In addition, a wound-healing assay was used to assess the migration in siE2F8-transfected ovarian cancer cells; E2F8-knockdown cells exhibited reduced migration (Figure 3B). Notably, even TOV112D, which showed no effect of siE2F8 on cell proliferation, exhibited the effects of E2F8 knockdown on cellular invasion and migration. Our findings imply that E2F8 expression is correlated with cancer invasion and metastasis.

### 2.4. E2F8 Knockdown Blocked Tumor Growth in Xenograft Nude Mouse Model

**Figure 4:**
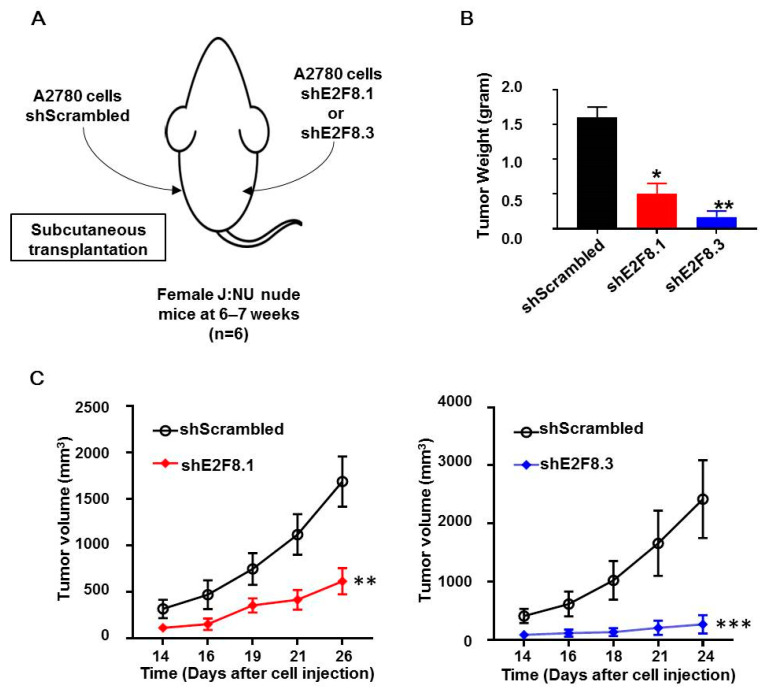
E2F8-knockdown decreases tumor size in xenograft nude mouse model. (**A**) A2780 cells transfected with shE2F8.1 or shE2F8.3 or shScrambled were injected subcutaneously into the right or left flanks of female J:NU nude mice at 6–7 weeks. (**B**) Tumor weights were compared after harvesting tumors. (**C**) Tumor sizes were serially measured and compared. * *p* < 0.05, ** *p* < 0.01, *** *p* < 0.001, vs. shScrambled.

The A2780 cells transfected with shE2F8.1 or shE2F8.3 or shScrambled were injected subcutaneously into the right or left flank of each of the six nude mice (Figure 4A). Then, tumor sizes were measured, and tumor growth was compared. The weight of tumors originating from the E2F8-knockdown cells was reduced, compared to that of the control tumors (Figure 4B). Furthermore, the tumors originating from the E2F8-knockdown A2780 cells showed a significantly reduced tumor size, compared to that of the control tumors (Figure 4C).

### 2.5. Effects of E2F8 Knockdown on EMT and Notch Pathways

**Figure 5:**
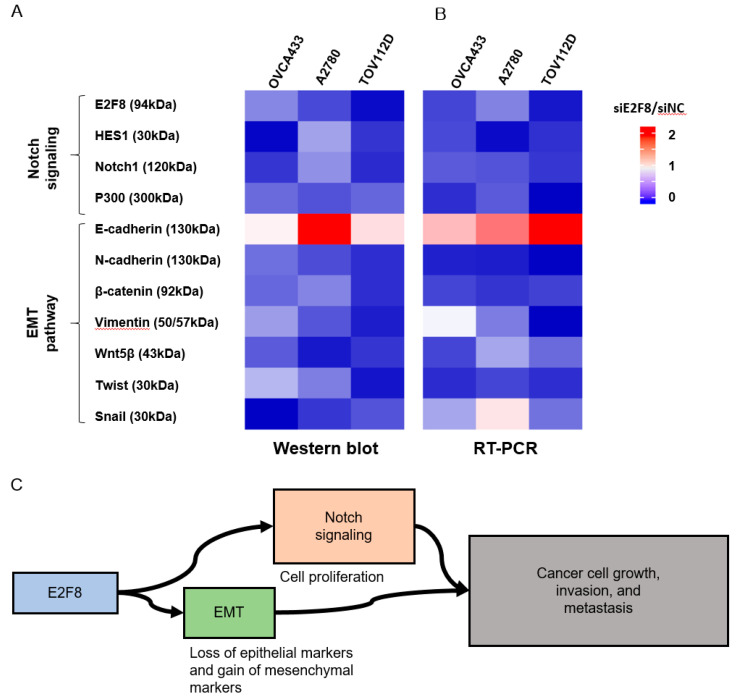
Effect of E2F8 knockdown on Notch and epithelial-mesenchymal transition (EMT) pathways. OVCA433, A2780, and TOV112D cells were transfected with siE2F8 and siNC for 48 h. Western blot (**A**) and Real-time RT-PCR (**B**) of Notch- and EMT-related genes after E2F8 knockdown in OVCA433, A2780, and TOV112D cells. The ratio of siE2F8- to siNC-treated expression in both protein and mRNA is presented in the heat map image. A ratio smaller than 1 meant that E2F8 knockdown decreased the amount of expression (blue). Conversely, a ratio greater than 1 meant that E2F8 knockdown increased the expression (red). (**C**) E2F8 promotes tumor growth, invasion, and metastasis through EMT and Notch signaling pathways. Data are the mean ± SD.

Because EMT is crucial in cell migration and invasion, the identification of factors associated with EMT may have clinical impacts [24,25]. Therefore, we examined whether E2F8 is associated with EMT. The EMT-related markers were evaluated using real-time RT-PCR and Western blotting after the siRNA-mediated knockdown of E2F8 in OVCA433, A2780, and TOV112D cells (Figure 5A,B). The ratio of siE2F8- to negative control siRNA (siNC)-treated expression in both protein and mRNA was presented in the heat map image. A ratio smaller than 1 meant that E2F8 knockdown decreased the amount of expression. Conversely, a ratio greater than 1 meant that E2F8 knockdown increased the expression. The knockdown of E2F8 resulted in an increase in E-cadherin expression as well as decreases in the expression of N-cadherin, β-catenin, vimentin, Wnt5β, and Twist. In addition, the EMT-mediating transcription factor Snail was downregulated in the siE2F8-transfected cells, compared to their pertinent levels in the siNC-transfected cells. These results indicate that the dysregulation of the EMT-related genes may explain the involvement of E2F8 in ovarian cancer cell migration and invasion.

The Notch signaling cascade is critical in cell proliferation, differentiation, development, and homeostasis. Deregulated Notch signaling is observed in various types of malignancies [26]. Additionally, to discover the underlying mechanism by which E2F8 promotes a malignant phenotype in ovarian cells, we assessed the status of critical signaling cascades controlled by Notch in the E2F8-knockdown cells (Figure 5A,B). The raw image of the Western blot is shown in Supplement Figure 2. The E2F8 knockdown in the OVCA433, A2780, and TOV112D cells resulted in the downregulation of NOTCH1, HES1, and p300 expression at the mRNA and protein levels. These data indicate that E2F8 mediates tumor growth, invasion, and affects the EMT and Notch signaling pathways (Figure 5C).

## 3. Disussion

In this study, we investigated the biofunctional role of E2F8 in epithelial ovarian cancer progression, and our results established a vital role for E2F8 as a promoter of ovarian cancer cell proliferation, migration, and invasiveness. Importantly, we demonstrated that the overexpression of E2F8 has clinically relevant prognostic significance in ovarian cancer patients. Furthermore, our results reveal a potential molecular mechanism by which E2F8 promotes cell proliferation and invasiveness in ovarian cancer upon activating the EMT and Notch signaling pathways (Figure 5C). Our findings provide substantial evidence that the upregulation of E2F8 plays an essential role in promoting ovarian cancer progression, and that E2F8 may represent a novel prognostic biomarker and therapeutic target for this disease.

Even though there is a high initial response rate to first-line platinum or taxane chemotherapy, most patients with ovarian cancer suffer from recurrences. Commonly diagnosed at an advanced stage, aggressively progressive characteristics have been identified as a major obstacle to the long-term remission of ovarian cancer, and an effective strategy is required for overcoming cancer progression, including migration and invasion [27]. In this study, we focused on E2F8 as a potential target for the inhibition of ovarian cancer progression.

The E2F family proteins have been shown to be crucial regulators of many processes relevant to malignancy. Recently identified, E2F8 has been shown to function as a potent cell cycle regulator and has emerged as a critical proliferation promoter in multiple malignancies [12,13,15]. Still, the biological role and clinical significance of E2F8 in epithelial ovarian cancer remained mostly unidentified. To the best of our knowledge, there have been only two reports on the roles of E2F family members in ovarian cancer patients [15,16]. However, these two studies focused on other E2Fs, including E2F1, E2F2, and E2F7. Thus, this is the first study to evaluate the potential of E2F8 as a therapeutic target for epithelial ovarian cancer treatment.

Conventionally, the E2F family proteins function as either activators (E2F1-3a) or repressors (E2F3b-8) of transcription during cell cycle regulation [5]. At first, E2F8 was thought to function as a repressor, because downregulated E2F-target genes were found to block cell cycle progression in fibroblasts [28,29]. Nonetheless, several contemporary studies have provided evidence supporting the theory that E2F family members can act as either transcriptional activators or repressors depending on the cellular and tissue context or the target genes involved [30,31,32,33]. Remarkably, E2F8 was reported to promote the transcriptional activation of *VEGFA* via hypoxia-inducible factor 1 while suppressing the transcription of *VEGFR1/2* in endothelial cells during embryonic and tumor development [10,34]. Likewise, E2F8 is thought to stimulate *CCBE1* promoter activity and suppress that of *FLT4,* which controls lymphangiogenesis in zebrafish embryonic development [11]. Additionally, E2F8 was shown to transcriptionally upregulate *UHRF1* in lung cancer and cyclin D1 in hepatocellular carcinoma [12,13]. Therefore, these recent findings suggest that E2F8 regulates a variety of downstream genes in a context-dependent manner. Herein, we demonstrated that E2F8 upregulates the EMT and Notch signaling pathways, and a further follow-up study is being planned to support the hypothesis that E2F8 promotes ovarian cancer progression by upregulating multiple cell cycle regulators.

The Notch signaling pathway contributes to tumor progression characteristics, including invasion, EMT, metastasis, and angiogenesis [22,35]. A previous study found that the expression of Notch gradually increased with the poor differentiation of ovarian cancer tissues and with the advancement of the FIGO stage in patients with epithelial ovarian cancer [36]. This finding supports the putative function of the Notch signaling pathway in tumor progression; however, there is little evidence of a regulatory correlation between the Notch-related downstream factors and E2F8.

Since many of these pathways have been studied only in transformed cell lines possessing abnormal E2F activity, it is necessary to further explore the relevance of many methods of modulating E2F in both cell proliferation and cancerous situations. Some of these regulations may vary from cancer to cancer and may provide new markers of cancer progression or opportunities for therapeutic intervention. Finally, there is cumulative evidence that enhanced E2F activity is an important mechanism used to develop chemo-resistance by cancer cells, especially for CDK4/6 inhibitors [37,38,39,40,41]. Thus, the targeted inhibition of E2F or targeted killing of transformed cells with tumor gene driven E2F activity would be an excellent complement to current treatment strategies.

Our research further demonstrated that E2F8 expression was significantly correlated with that of the EMT and Notch signaling pathway-related markers, indicating that E2F8 is among the factors influencing tumor progression through the EMT and Notch signaling pathways. Furthermore, the clinical outcome data from 115 patients with epithelial ovarian cancer supported the findings of our in vitro experiments.

## 4. Methods

### 4.1. Patients and Tissue Samples

A total of 115 ovarian cancer tissue samples were obtained from patients with epithelial ovarian cancer at the time of staging the operation. Patients with borderline ovarian tumor or concomitant gynecological or other primary malignancy, as well as those who had received preoperative neoadjuvant chemotherapy, were excluded. The median period of follow-up was 52 months (range: 2–136 months) for survivors. Progression-free survival was defined as the interval between the date of surgery and the date of progression as confirmed by imaging. The control group consisted of 60 normal ovarian epithelial tissue samples obtained from patients who underwent simple hysterectomy with oophorectomy for benign uterine conditions. We froze all tissue samples immediately in liquid nitrogen and stored them at –80 °C.

### 4.2. Ethics Approval and Consent to Participate

This study was approved by the Institutional Review Board (IRB) of Severance Hospital, Yonsei University College of Medicine (No.: 2020-0018-001). The need for informed consent was waived because of the low risk designated by the IRB. All procedures were approved by the Institutional Animal Care and Use Committee of Yale University and were compliant with the legal mandates and federal guidelines for the care and maintenance of laboratory animals.

### 4.3. Tumor Microarray (TMA) Construction and Immunohistochemistry (IHC)

Formalin-fixed, paraffin-embedded tissues from 66 cases of human ovarian cancer were used. The most representative tumor area without necrosis was carefully selected and marked on hematoxylin- and eosin-stained slides. The formalin-fixed, paraffin-embedded tissues corresponding to the marked areas were sampled using a commercially available TMA instrument (AccuMax Arrayer, Petagen, Inc., Seoul, Korea).

The protein expression of E2F8 was immunohistochemically examined using the REAL EnVision Detection System (Dako, Glostrup, Denmark) in accordance with the manufacturer’s instructions. Briefly, 4 µm sections of TMA tissue blocks were deparaffinized with Bond Dewax Solution (Vision BioSystems, Mount Waverly, VIC, Australia), and an antigen retrieval procedure was performed using Bond ER Solution (Vision BioSystems) for 30 min at 100 °C. Endogenous peroxidases were quenched by incubation with hydrogen peroxide for 5 min. The sections were incubated for 15 min at ambient temperature with a mouse monoclonal anti-E2F8 antibody (clone 3E9-2F5, Novus Biologicals, Littleton, CO, USA).

The IHC staining of E2F8 was scored semiquantitatively. The percentage of E2F8-positive tumor cells was classified into one of four categories: 1, <25%; 2, 25%–49%; 3, 50%–74%; and 4, ≥75%. The staining intensity in the tumor cells was scored on a scale of 0–3: 0, negative; 1, weak; 2, moderate; and 3, strong. We calculated the final score by multiplying the intensity score and percentage score. The immunoreactivity of E2F8 was then classified as Fnegative (score 0), weak (score 1–6), or positive (score 8–12) expression. Negative or weak E2F8 immunoreactivity was considered as low E2F8 expression, and positive E2F8 immunoreactivity was considered as high E2F8 expression.

### 4.4. Cell Lines

We obtained the SKOV3 human epithelial ovarian cancer cell line from the Korean Cell Line Bank (KCLB, Seoul, Korea) and purchased the A2780 cell line from the European Collection of Cell Cultures (ECACC, Wiltshire, UK). The OVCA433 and TOV112D ovarian cancer cell lines were provided by the Korea Gynecologic Cancer Bank. The human ovarian surface epithelial (HOSE) cells were obtained from ScienCell Research Laboratories (San Diego, CA, USA). The SKOV3 cell line was derived from a 64 year old Caucasian patient with ovarian cancer [42]. The A2780 ovarian cancer cell line was established from the tumor tissue of an untreated patient [43]. The OVCA433 cell was originally isolated from ovarian cancer patient ascites [44]. The TOV112D cell line was initiated in October of 1992 from a patient of French-Canadian descent with early onset ovarian cancer and an unknown family history of ovarian cancer [45]. The subtypes of the epithelial ovarian cancer cell lines are as follows: OVCA433, serous; TOV112D, endometrioid; SKOV3, serous; and A2780, nonspecified [46].

The SKOV3 and A2780 cells were cultured in RPMI-1640 medium (Gibco-BRL, Gaithersburg, MD, USA), whereas the OVCA433 and TOV112D cells were cultured in Dulbecco’s modified Eagle’s medium (Gibco-BRL, Grand Island, NY, USA). The HOSE cells were cultured in ovarian epithelial cell medium (OEpiCM, ScienCell, Carlsbad, CA, USA). All culture media were added with 10% (vol/vol) fetal bovine serum (FBS, Sigma Aldrich, St. Louis, MO, USA) and 1% penicillin or streptomycin (Sigma Aldrich, St. Louis, MO, USA), and the cell lines were maintained at 37 °C in a humidified atmosphere of 5% CO_2_ and 95% air. Each culture medium was replaced with a fresh medium every 2–3 days, and the cells were used between passages 5 and 10.

### 4.5. Small Interfering RNA (siRNA) Transfection

The *E2F8-*targeting siRNA (siE2F8) and negative control siRNA (siNC) were obtained from Genolution (Genolution Pharmaceuticals Inc., Seoul, Korea). Cells (5 × 10^4^ cells/well) were allocated into six-well plates and transfected with 10 nM siRNA in phosphate-buffered saline using the G-Fectin Kit (Genolution Pharmaceuticals) in accordance with the manufacturer’s protocol. The siRNA-transfected cells were used in the assays in vitro 48 h after transfection. The experiments were repeated at least three times. The target sequence of siE2F8 is shown in Supplementary Table S2.

### 4.6. Construction of Lentiviral E2F8-shRNA Vector

The sequences of shRNAs targeting *E2F8* (shE2F8.1 and shE2F8.3) were 5′-GCCGCAAAGACAAGTCTTTAA-3′ (TRCN0000017428) and 5′-CGCCGAGCAGATTATGATGAT-3′ (TRCN0000017430). The HEK293T cells were transfected with shRNA-encoding lentiviral vector DNA and packaging vectors (pCMV-VSVG and ∆8.91) using Lipofectamine 2000 (Invitrogen, Carlsbad, CA, USA). A viral medium was collected at both 48 and 72 h post-transfection. The cells were infected using the viral medium along with 8 μg/mL polybrene (Sigma Aldrich), followed by selection with puromycin (Sigma Aldrich). Doxycycline-inducible shE2F8.1 and shE2F8.3 vectors were purchased (Vigene Biosciences, Rockville, MD, USA), and the stable transfected A2780 cell line was established. After treatment with doxycycline (Sigma Aldrich), protein lysates were obtained daily from day 1 to 5, and E2F8 expression was assessed. The proliferation of doxycycline-inducible shE2F8.1- or shE2F8.3-expressing stable A2780 cell lines in the presence or absence of doxycycline was also assayed.

### 4.7. Quantitative Real-Time PCR (qRT-PCR)

RNA was extracted from cancer tissues or cultured cells using TRIzol reagent (Invitrogen). Total RNA was reverse-transcribed into cDNA using a Reverse Transcription Reagent Kit (Invitrogen) according to the manufacturer’s protocols. Real-time PCR analyses were conducted employing a SYBR Green Real-time PCR Kit (TOYOBO Co. Ltd., Osaka, Japan). The amplification of *E2F8* involved the following settings: initial denaturation at 95 °C for 3 min, followed by 40 cycles of denaturation at 95 °C for 15 s, annealing at 60 °C for 60 s, elongation at 72 °C for 60 s, and a final elongation at 72 °C for 5 min. The qRT-PCR was accomplished using an ABI StepOnePlus Real-Time PCR System (Applied Biosystems, Foster City, CA, USA). The obtained data were normalized to the expression of U6 and GAPDH. Primer and probe sequences are available upon request. The relative change in the expression of mRNA was calculated using the 2–ΔΔCT method. All qRT-PCR experiments were replicated at least three times. The PCR primers used are shown in Supplementary Table S3.

### 4.8. Cell Proliferation Assay

A Cell Counting Kit-8 (CCK-8) assay (Dojindo Laboratories, Kumamoto, Japan) was used to assess cell proliferation. Cells (2 × 10^3^ cells/well) were allocated into 96-well flat-bottomed plates in 100 μL complete medium. The cells were incubated overnight for cell attachment and recovery. Subsequently, these cell were transfected with siNC or siE2F8 for 24, 48, or 72 h. An aliquot of 10 µL CCK-8 solution was added to each well and incubated for 2 h. The absorbance was measured at 450 nm to calculate the number of viable cells in each well. The assay was performed in triplicate.

CellTiter-Glo assay (Promega, Madison, WI, USA) was performed to compare the cell proliferation between shE2F8- and shScrambled-transfected cells. Briefly, the transfected cells (2 × 10^3^ cells/well) were allocated into five 96-well flat-bottomed plates in 100 μL complete medium. The plates were allowed to equilibrate at room temperature for 30 min. Equal volumes of CellTiter-Glo reagents were added directly to the wells. The plates were incubated at room temperature for 10 min on a shaker, and the fluorescence was measured with a luminometer. Luminescence readings were normalized to and expressed as a relative percentage of the plate-averaged vehicle-treated control.

### 4.9. Clonogenic Survival Assay

At 48 h after transfection with the indicated shRNAs, the 2 × 10^3^ cells were transferred to six-well plates. The culture medium was changed at 4 or 5 days for another 2 weeks. The medium was removed, and the colonies were fixed with 10% formalin for 15 min, followed by staining with crystal violet for visualization. Colonies larger than 0.5 mm in diameter were counted using ImageJ software (NIH Bethesda, MD, USA). For each treatment group, each well was assessed in triplicate.

### 4.10. Wound-Healing Migration Assay

Cell migration was evaluated using a wound-healing assay. About 5 × 10^5^ cells were allocated into 6-well culture plates with a serum-containing medium and were allowed to grow to 90% confluence in a complete medium. The serum-containing medium was removed, and the cells were serum-starved for 24 h. At 100% confluence, an artificial, homogenous wound was made by scratching the monolayer using a sterile 200 μL pipette tip. Following the scratching, the cells were washed with serum-free medium. Pictures of cells migrating into the wound were captured at 0, 12, 24, 36, and 48 h using a microscope. Three independent experiments were performed in triplicate.

### 4.11. Matrigel Invasion Assay

We performed a Matrigel invasion assay with the BD Biocoat Matrigel Invasion Chamber (pore size: 8 mm, 24-well; BD Biosciences, Bedford, MA, USA), in accordance with the manufacturer’s protocol. A total of 5 × 10^4^ cells was seeded in the upper chamber in a serum-free medium, and a complete medium was added to the bottom chamber. The Matrigel invasion chamber was incubated at 37 °C under 5% CO_2_ for 48 h. Noninvading cells were eliminated from the upper chamber with cotton-tipped swabs. The cells that had invaded the lower side of the filter through the pores were stained (Diff Quik, Sysmes, Kobe, Japan) and were counted with a hemocytometer. We repeated this assay at least three times.

### 4.12. Western Blotting Analysis

A radioimmunoprecipitation assay (RIPA) buffer (Thermo Fisher Scientific Inc., Waltham, MA, USA) was used to extract proteins. We measured the protein concentrations using the Pierce BCA Protein assay kit (Thermo Fisher Scientific). The proteins were boiled with 2× sample buffer and were subsequently resolved on 10% sodium dodecyl sulfate-polyacrylamide gels before being electrophoretically transferred to polyvinylidene difluoride membranes (Millipore, Billerica, MA, USA). After blocking with 5% nonfat dried milk in 1× Tris-buffered saline containing 0.1% Tween 20 (pH 7.6) (TBST) at room temperature for 1 h, the membranes were incubated with anti-β-catenin, anti-Notch1, anti-HES1, anti-P300, anti-E-cadherin, anti-N-cadherin, anti-Vimentin, anti-Snail, (all from Cell Signalling Technologies, Danvers, MA, USA), anti-E2F8, anti-Wnt5β, and anti-Twist (all from Abcam, Cambridge, MA) antibodies overnight at 4 ℃. Anti-β-actin antibody (Sigma-Aldrich) was used as an internal control. Membranes were washed with TBST and incubated with horseradish peroxidase-conjugated secondary antibodies (Jackson Immunoresearch, West Grove, PA, USA) for 1 h at room temperature. After washing again with TBST, signal was detected using an enhanced chemiluminescence kit (Thermo Scientific, Rockford, IL, USA), and intensity was quantified using ImageJ software.

### 4.13. In-Vivo Experiment

We obtained Female J:NU nude mice from Jackson Laboratory (Bar Harbor, ME, USA) which were used at 6–7 weeks of age. Mice were kept in aseptic conditions with a constant temperature and humidity (Yale University protocol). The A2780 cells were pretreated with shE2F8.1, shE2F8.3, and shScrambled for 24 h, followed by subcutaneous transplantation (1.0 × 10^6^ cells/flank, xenograft *n* = 6, 7–8 weeks of age). All xenografts were transplanted into both the right and left dorsal flanks of the mice. Tumor size was measured with digital calipers, and the volume was calculated using the formula 0.52 × length × width^2^. The mice were sacrificed using a carbon dioxide chamber at the end of the study.

### 4.14. Statistical Analysis

The IBM SPSS version 23 for Windows (SPSS Inc., Chicago, IL, USA) and the open-source program R v3.5.1 (ISBN 3–900,051–07-0, http://www.R-project.org; R Foundation for Statistical Computing, Vienna, Austria) were used in the statistical analysis. The Kolmogorov–Smirnov test was used to validate the standard normal distribution assumptions. Pearson’s chi-square test, Fisher’s exact test, Student’s *t*-test, and the Mann–Whitney *U*-test were used for univariate analysis. Survival outcomes were determined using the Kaplan–Meier survival analysis. Univariate and multivariate analyses of the effects of various prognostic factors on survival were performed using the Cox proportional hazards model. Multivariate analysis was performed with variables that were considered significant in the univariate analysis. A *P*-value of less than 0.05 was regarded as statistically significant.

## 5. Conclusions

Overall, our results indicate that E2F8 enhances ovarian cancer cell proliferation, migration, and invasion, probably through the EMT and Notch signaling pathways. These findings further suggest that E2F8 is a promising prognostic marker and therapeutic target against ovarian cancer.

## Figures and Tables

**Table 1:** Univariate and multivariate analyses of various factors correlated with progression-free survival.

	No. of Patients	Progression-Free Survival			
		Univariate Analysis		Multivariate Analysis	
		HR (95% CI)	*p*	HR (95% CI)	*p*
Age, years (continuous)	115	0.996 (0.975–1.017)	0.683		
FIGO stage					
I, II	11	1.0		1.0	
III, IV	104	5.519 (1.318–23.1.6)	0.019	5.361 (1.293–22.224)	0.021
Tumor grade					
Low	4	1.0			
High	111	3.614 (0.484–26.996)	0.21		
Preoperative CA 125, U/mL					
≤500	51	1.0			
>500	64	1.004 (0.620–1.625)	0.987		
Residual disease					
NGR	75	1.0		1.0	
R < 1 cm	40	17.787 (8.400–37.664)	<0.001	19.029 (9.030–40.100)	<0.001
E2F8 expression					
Low	34	1.0		1.0	
High	81	1.887 (1.073–3.318)	0.028	1.590 (1.010–2.500)	0.015

HR, hazard ratio; FIGO, International Federation of Gynecology and Obstetrics; CA, cancer antigen; CI, confidence interval; NGR, no gross residual

## Supplementary Materials

Supplementary Materials can be found at https://www.mdpi.com/1422-0067/21/16/5813/s1.

## Abbreviations

**E2F8**	**E2F transcription factor 8**
EMT	epithelial–mesenchymal transition
TMA	tumor microarray
IHC	immunohistochemistry
KCLB	Korean Cell Line Bank
ECACC	European Collection of Cell Cultures
HOSE	human ovarian surface epithelial cells
OEpiCM	varian epithelial cell medium
siRNA	small interfering RNA
siE2F8	*E2F8-*targeting siRNA
siNC	negative control siRNA
shE2F8.1 and shE2F8.3	shRNAs targeting *E2F8*
qRT-PCR	quantitative real-time PCR
CCK-8	Cell Counting Kit-8
RIPA	radioimmunoprecipitation assay
FIGO	International Federation of Gynecology and Obstetrics
NRF	National Research Foundation of Korea
HR	hazard ratio
CI	confidence interval
CA	cancer antigen
NGR	no gross residual;
