# Supplementary material for: E2F8 Induces Cell Proliferation and Invasion through the Epithelial–Mesenchymal Transition and Notch Signaling Pathways in Ovarian Cancer
Source: Int J Mol Sci. 2020 Aug 13;21(16):5813. doi: 10.3390/ijms21165813 (PMC7460858; doi:10.3390/ijms21165813)

Supplementary Table 1. Patient characteristics

**Variables**	E2F8 expression		P value
	Low (n=34)	High (n=81)	
Age, year mean [range]	56.02 [35-70]	54.08 [30-83]	0.39
FIGO stage			
1	2 (5.9)	4 (4.9)	0.236
2	2 (5.9)	3 (3.7)	
3	24 (70.6)	44 (54.3)	
4	6 (17.6)	30 (37.0)	
Histology			
Serous	34 (100)	81 (100)	> 0.99
Grade			
Low	3 (8.8)	1 (1.2)	0.156
High	31 (91.2)	80 (98.8)	
CA 125, U/mL mean [range]	2334.32 [7-12000]	1665.07 [8-10614]	0.247
Residual disease			
NGR	25 (73.5)	50 (61.7)	0.285
R <1 cm	9 (26.5)	31 (38.3)	

FIGO, The International Federation of Gynecology and Obstetrics; NGR, no gross residual disease; R, residual disease

Supplementary Table 2. *E2F8* siRNA sequence

	Sequence 5′-3′
E2F8 siRNA sequence	Sense: CAAGAAUAGAGUCUGUUAAUU<br>Anti-sense: UUAACAGACUCUAUUCUUGUU

Supplementary Table 3. PCR primer sequences (5′-3′)

	Forward	Reverse
E2F8	CCACCACAGCAAATATCGTG	CTTTGGCCTCAGGTAATCCA
HES1	TCAACACGACACCGGATA AA	TCAGCTGGCTCAGACTTTCA
Notch1	GCCGCCTTTGTGCTTCTGTTC	CCGGTGGTCTGTCTGGTCGTC
P300	GACCCTCAGCTTTTAGGAATCC	TGCCGTAGCAACACAGTGTCT
E-cadherin	ATTCTGATTCTGCTGCTCTTG	AGTAGTCATAGTCCTGGTCCT
N-cadherin	CCCAAGACAAAGAGACCCAG	GCCACTGTGCTTACTGAATTG
ß-catenin	TGCAGTTCGCCTTCACTATG	ACTAGTCGTGGAATGGCACC
Vimentin	TGGATTCACTCCCTCTGGTT	GGTCATCGTGATGCTGAGAA
Wnt5ß	TGTGAGGTGAAGACCTGCTG	AAAGTTGGGGGAGTTCTCGT
Twist	CGGGAGTCCGCAGTCTTA	TGAATCTTGCTCAGCTTGTC
Snail	GAGGCGGTGGCAGACTAG	GACACATCGGTCAGACCAG
U6	CTCGCTTCGGCAGCACA	AACGCTTCAGGAATTTGCGT
GAPDH	TCGACAGTCAGCCGCATCTTCTTT	ACCAAATCCGTTGACTCCGACCTT

Supplementary Figure 1. Using siE2F8, E2F8 knockdown was performed in the E2F8-elevated cell lines.

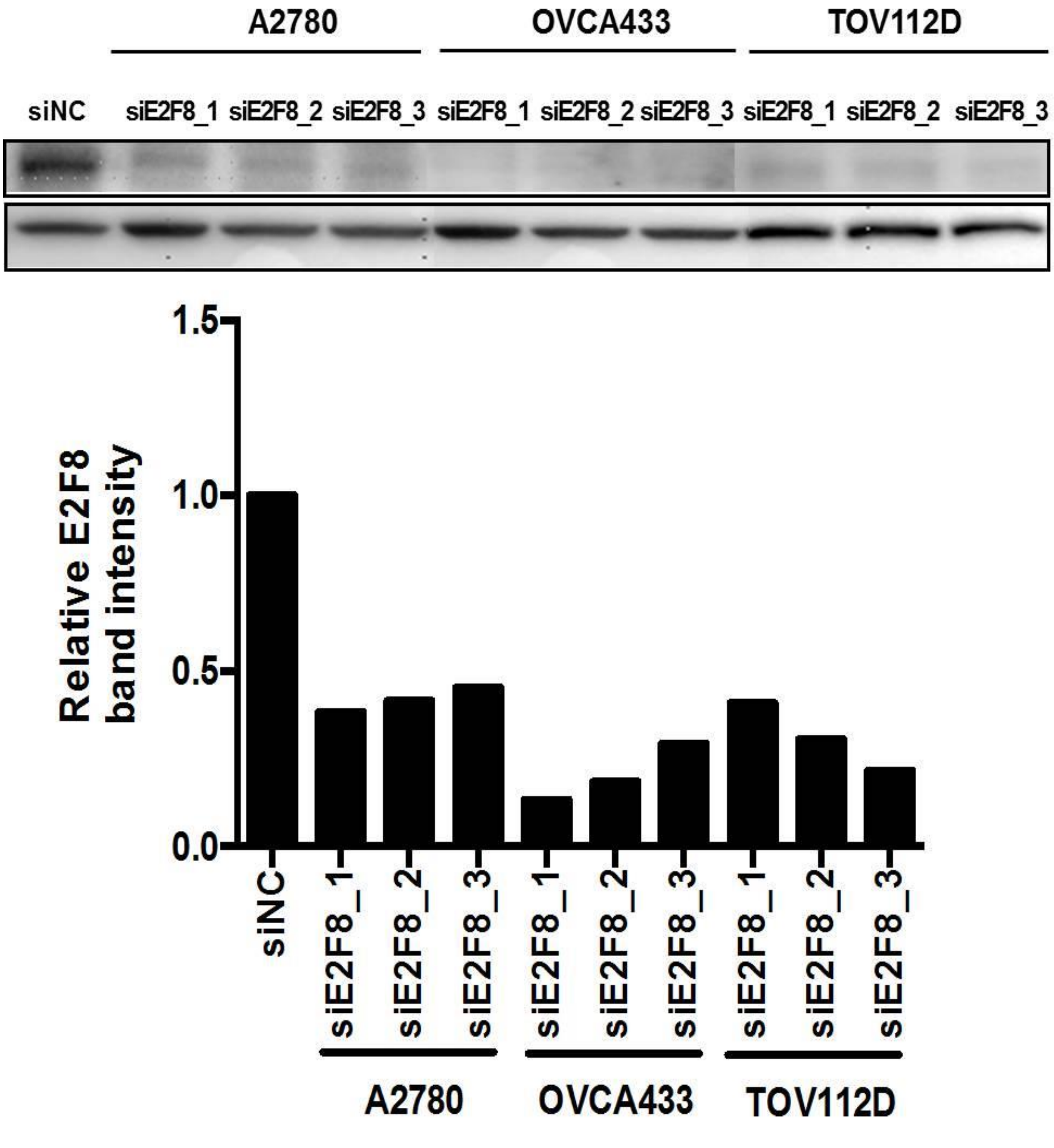

Supplementary Figure 2. Eeffect of E2F8 knockdown on Notch and EMT pathways in OVCA433, A2780, and TOV112D cells

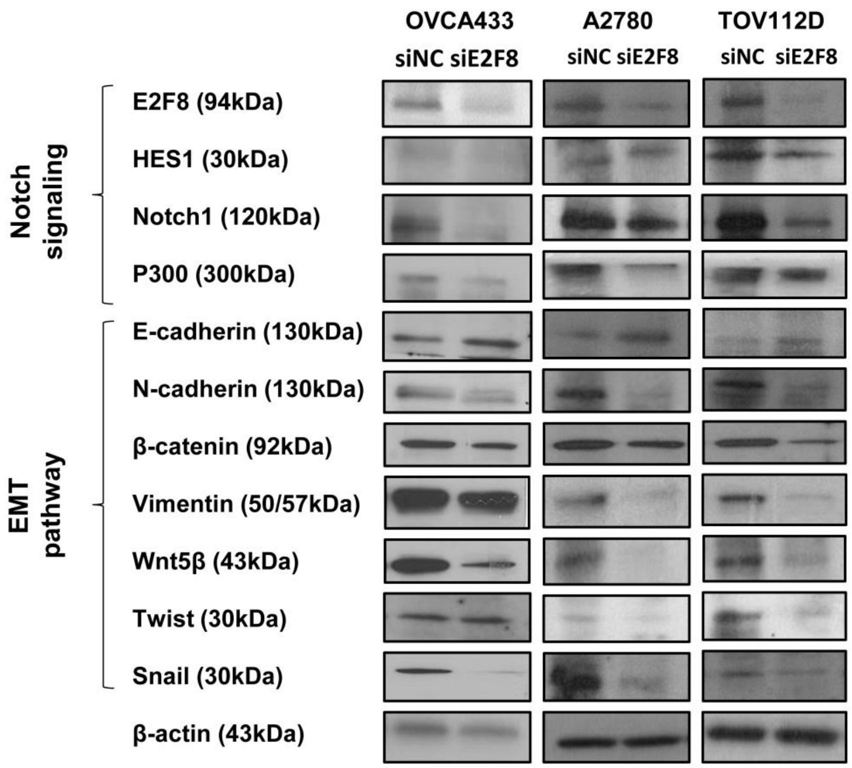

Supplement: Supplementary file 1 [file ijms-21-05813-s001.pdf]
